# In vitro assessment of a computer-designed potential anticancer agent in cervical cancer cells

**DOI:** 10.1186/s40659-016-0104-5

**Published:** 2016-11-02

**Authors:** Michelle Helen Visagie, Seema Rummurat Jaiswal, Anna Margaretha Joubert

**Affiliations:** Department of Physiology, University of Pretoria, Private Bag X 323, Arcadia, Pretoria, 0007 South Africa

**Keywords:** Morphology, Apoptosis, Cervical cancer, ESE-15-ol

## Abstract

**Background:**

Computer-based technology is becoming increasingly essential in biological research where drug discovery programs start with the identification of suitable drug targets. 2-Methoxyestradiol (2ME2) is a 17β-estradiol metabolite that induces apoptosis in various cancer cell lines including cervical cancer, breast cancer and multiple myeloma. Owing to 2ME2’s poor in vivo bioavailability, our laboratory in silico-designed and subsequently synthesized a novel 2ME2 analogue, 2-ethyl-3-*O*-sulphamoyl-estra-1,3,5(10),15-tetraen-17-ol (ESE-15-ol), using receptor- and ligand molecular modeling. In this study, the biological effects of ESE-15-ol (180 nM) and its parent molecule, 2ME2 (1 µM), were assessed on morphology and apoptosis induction in cervical cancer cells.

**Results:**

Transmission electron microscopy, scanning electron microscopy and polarization-optical transmitted light differential interference contrast (PlasDIC) images demonstrated morphological hallmarks of apoptosis including apoptotic bodies, shrunken cells, vacuoles, reduced cell density and cell debris. Flow cytometry analysis showed apoptosis induction by means of annexin V-FITC staining. Cell cycle analysis showed that ESE-15-ol exposure resulted in a statistically significant increase in the G_2_M phase (72%) compared to 2ME2 (19%). Apoptosis induction was more pronounced when cells were exposed to ESE-15-ol compared to 2ME2. Spectrophotometric analysis of caspase 8 activity demonstrated that 2ME2 and ESE-15-ol both induced caspase 8 activation by 2- and 1.7-fold respectively indicating the induction of the apoptosis. However, ESE-15-ol exerted all of the above-mentioned effects at a much lower pharmacological concentration (180 nM) compared to 2ME2 (1 µM physiological concentration).

**Conclusion:**

Computer-based technology is essential in drug discovery and together with in vitro studies for the evaluation of these in silico-designed compounds, drug development can be improved to be cost effective and time consuming. This study evaluated the anticancer potential of ESE-15-ol, an in silico-designed compound in vitro. Research demonstrated that ESE-15-ol exerts antiproliferative activity accompanied with apoptosis induction at a nanomolar concentration compared to the micromolar range required by 2ME2. This study is the first study to demonstrate the influence of ESE-15-ol on morphology, cell cycle progression and apoptosis induction in HeLa cells. In silico-design by means of receptor- and ligand molecular modeling is thus effective in improving compound bioavailability while preserving apoptotic activity in vitro.

## Background

Cervical cancer is a leading cause of death for women worldwide [1]. Antimitotic compounds are still one of the most widely used chemotherapeutic drugs for cervical cancer treatment. Chemotherapeutic drugs include agents that target the cell cycle resulting in antiproliferative activity and subsequent apoptosis induction [2, 3].

Microtubule-targeting agents are divided into two traditional categories: microtubule-destabilizing agents such as the vinca alkaloids vinblastine, vincristine and colchicine and microtubule-stabilizing agents such as the taxanes (paclitaxel and docetaxel) [4]. Agents that bind to the colchicine-binding site of microtubules are in various stages of clinical trials and include combretastatins, as well as 2-methoxyestradiol (2ME2) [5]. Microtubules are essential for cytoskeleton integrity, embryonic development, cell division, intracellular molecular transport system, gene activation and potentially play a role in cervical cancer treatment [6].

2ME2 is a naturally occurring 17β-estradiol metabolite synthesized from sequential hepatic hydroxylation and methylation by cytochrome P450 enzymes and catechol-*O*-methyltransferase [7]. 2ME2 exerts estrogen-independent activity by targeting the microtubule skeleton and by inhibiting tumour growth and angiogenesis. In addition, 2ME2 inhibits the tubulin polymerization in vitro, thus disrupting normal microtubule functioning [7]. 2ME2 induces apoptosis by activating the intrinsic- and extrinsic cascade by both caspase dependent- and independent pathways [4]. In addition to the inhibition of microtubule dynamics and induction of cell cycle arrest, the pro-apoptotic activity of 2ME2 has also been associated with stimulation of cellular reactive oxygen species (ROS) production, resulting in the release of cytochrome *c* from the mitochondria and activation of caspases [7].

In vitro, 2ME2 exerts antiproliferative activity in a wide variety of cell lines including tumorigenic estrogen receptor positive MCF-7 breast cancer cells, cervical cancer HeLa cells and HCT116 colon cancer cells [7, 8]. 2ME2 (Panzem^®^) was evaluated in several phase I and II clinical trials [5]. Therapeutic doses in vivo resulted in a metaphase arrest, and, as a consequence, inhibition of cell proliferation and induction of apoptosis [7]. Results from preclinical tumor models in animals suggest that, maintaining a plasma concentration of 2ME2 in the range of 10–56 nM, is required for efficient antitumor activity [9, 10].

Undesirable biopharmaceutical properties including rapid metabolism and low bioavailability of 2ME2 resulted in identification of estradiol analogues with improved in vivo efficacy. 2ME2 is metabolized with conjunction at the 3- and 17 positions, together with oxidation at the 17 position [11]. Structure–activity relationships indicated that the addition of a sulfamate group at position 3 of the estradiol backbone improves bioavailability of estradiol analogues and overcomes rapid biodegradation. This improves bioavailability since the sulphamate groups are highly reversible inhibitors of carbonic anhydrase II [5, 10]. The latter is most likely responsible for the high bioavailability of the sulphamoylated analogues since reversible uptake by erythrocytes and interaction with carbonic anhydrase II ensures transiting the liver without undergoing first pass metabolism [5].

It is well-recognised that drug discovery and subsequent development is a timely- and expensive process. Computer-based technology that combines chemical- and biological parameters in order to optimize drug identification, development and synthesis. Therefore, computer-based methods for identifying potential anticancer compounds are becoming increasingly important. These chemico-biological approaches in computer-based techniques used in drug discovery are referred to as in silico methods. Commonly used computational approaches include ligand-binding drug design, structure-based drug design (docking studies) and structure-activity relationships [12]. By means of docking studies and structure-activity relationships our laboratory in silico-designed several 17β-estradiol analogues where structure-activity relationships indicate enhanced potency and improved bioavailability [3, 5]. This study involved one of these novel compounds namely 2-ethyl-3-*O*-sulphamoyl-estra-1,3,5(10),15-tetraen-17-ol (ESE-15-ol). Docking studies of ESE-15-ol suggest that the selectivity is due to the double bond in the D-ring that is capable of interacting with histone 61 of the cancer associated carbonic anhydrase (CA) IX [3].

CAIX protein expression can be induced in MDA-MB-231 cells by the iron chelator, desferrioxamine mesylate (DFO) in a manner that results in extracellular acidification due to CAIX over-expression [13]. Reports indicate that ESE-15-ol prevents extracellular acidification in MDA-MB-231 cells due to CAIX expression, suggesting that the compound has the potential to curtail metastatic processes associated with acidotic micro-environmental conditions in tumours [3, 13, 14].

Previous in vitro crystal violet studies conducted in our laboratory revealed optimal antiproliferative activity exerted by sulphamoylated estradiol derivatives in nanomolar concentration range (110–220 nM) in breast tumourigenic cell lines (MDA-MB-231 and MCF-7), an oesophageal cancer cell line (SNO) and the cervical cancer HeLa cell line used in this study [5]. In addition, Stander et al. [3] reported that estradiol sulphamoylated derivatives inhibited carbonic anhydrase II activity between 167 and 180 ± 10 nM [3]. Since these sulphamoylated compounds are also hypothesized to be transported in the blood bound to carbonic anhydrase II, their influence on morphology of erythrocytes is noteworthy. Ex vivo exposure of 180 nm ESE-15-ol to whole blood for 24 h revealed no significant erythrocyte morphological damage and support future potential for in vivo studies [15].

In this study, the potential anticancer activity of ESE-15-ol, a computer-designed potential anticancer agent was assessed at a pharmacological concentration (180 nM) and compared to 2ME2 (1 µM, physiological concentration) on cell morphology, cell cycle progression and caspase activation in a cervical cancer cells. This merits the efficacy effectiveness of in silico-designed potential anticancer compounds for antiproliferative-, antimitotic- and apoptotic activity in future development of chemotherapeutic agents to be considered as an antitumour agents.

## Materials and methods

### Reagents and materials

The human epithelial cervical cell line (HeLa) was purchased through Sterilab Services (Johannesburg, South Africa) from American Tissue Culture Collection (ATCC) (Maryland, USA). The HeLa cell line is the oldest and utmost circulated immortalized cell line that presents with aggressive growth and is derived from cervical adenocarcinoma [16, 17]. HeLa cells were obtained from Hendrietta Lacks, a cervical cancer patient in 1951. The HeLa cell line is immortal and can divide an infinite number of times, is aneuploidy with 82 chromosomes and is infected with human papillomavirus 18 [18, 19].

All the required reagents of cell culture analytical grade were purchased from Sigma (St. Louis, USA) unless otherwise specified. Sterile cell culture flasks and plates were purchased from Sterile lab Services (Kempton Park, Johannesburg, South Africa). Penicillin, streptomycin, and fungizone were obtained from Highveld Biological (Pty) Ltd. (Sandringham, South Africa). The Annexin V fluorescein isothiocyanate (FITC) kit was purchased from BIOCOM biotech (Pty) Ltd. (Clubview, South Africa). 2ME2 was purchased from Sigma Chemical Co. (St. Louis, MO, USA). ESE-15-ol was synthesized by iThemba Pharmaceuticals (Pty) Ltd (Modderfontein, Gauteng, South Africa) since compound is not commercially available [5]. Aqueous osmium tetroxide, glutaraldehyde, phosphate buffer, quetol, Reynolds’ lead citrate, aqueous uranyl acetate and gold were supplied by the Electron Microscopy Unit of the University of Pretoria from Merck Co. (Munich, Germany). The Cryo-Scanning electron microscope (JEOL 840 with Cryostage) and the JOEL JEM 2100F transmission electron microscope (Electron Microscopy unit, University of Pretoria, South Africa) was used for viewing the prepared samples.

## Methods

### Cell culture

Cells were grown and maintained in 25 cm^2^ tissue culture flasks in a humidified atmosphere at 37 °C, 5% carbon dioxide (CO_2_) in a Forma Scientific water-jacketed incubator (Ohio, United States of America). HeLa cells were routinely cultivated in Dulbecco’s Minimum Essential Medium Eagle (DMEM) and supplemented with 10% heat-inactivated fetal calf serum (FCS) (56 °C, 30 min), 100 U/ml penicillin G, 100 μg/ml streptomycin, and fungizone (250 μg/ml). A stock solution of 2ME2 (2 μM) and ESE-15-ol (10,000 μM) compound dissolved in dimethyl sulphoxide (DMSO) were prepared. Both were subsequently diluted with medium to the desired concentrations before exposure of the cells. The medium of control cells was supplemented with an equal volume of DMSO (vehicle-treated control). The DMSO content of the final dilutions never exceeded 0.024% (v/v). Experiments were conducted in 6-well plates or 25 cm^2^ cell culture flasks.

For 6-well plates, exponentially growing cells were seeded at 350,000 cells per well in 3 ml maintenance medium on heat-sterilized coverslips. For 25 cm^2^ cell culture flasks, exponentially growing HeLa cells (1,000,000) were seeded in 25 cm^2^ flask with a final volume of 5 ml maintenance medium. After 24 h incubation at 37 °C to allow for cell adherence, medium was discarded and cells were exposed to ESE-15-ol and 2ME2 at the concentrations 180 nM and 1 µM, respectively. ESE-15-ol concentration of 180 nM was selected since previous studies conducted in our laboratory established optimal antiproliferative activity at this dosage (data not shown). A 2ME2 concentration of 1 µM was chosen as a previous study demonstrated antiproliferative activity the estrogen receptor positive breast epithelial adenocarcinoma MCF-7 cell line [10]. The vehicle control sample composed of DMSO and growth medium and the DMSO content of the final dilutions never exceeded 0.05% (v/v) and was exposed for 24 h for all the experiments.

### Polarization-optical transmitted light differential interference contrast

Polarization-optical transmitted light differential interference contrast (PlasDIC) is a method where the cells are illuminated with non-polarized light rendering improved contrast and a clear image [17, 20–22]. PlasDIC provides reproducibly high-quality imaging of individual cells, cell clusters creating a relief-type impression regardless of the of the plastic cell culture vessels thickness compared to the glass-bottom vessels required for differential interference contrast techniques [17, 18]. Images were captured before and after the exposure by means of the Axiovert 40 CFL microscope (Carl Zeiss, Gottingen, Germany).

### Transmission electron microscopy

Transmission electron microscopy was used to evaluate the influence of ESE-15-ol on the internal cellular structure. Cells were seeded (1,000,000) in a 25 cm^2^ flask with an overnight attachment policy [23]. Subsequently, medium was discarded and cells were exposed to the compounds. After 24 h, cells were trypsinized and resuspended in 1 ml medium. Cells were then fixed with 2.5% glutaraldehyde in 0.075 M phosphate buffer for 1 h, rinsed thrice with 0.075 M phosphate buffer, fixed with osmium tetroxide for 30 min, rinsed thrice with distilled water and dehydrated with increasing ethanol concentrations (30, 50, 70, 90, and 100%) [23]. Cells were then infiltrated with 50% quetol in ethanol for 1 h and then with 100% quetol for 4–6 h. Ultra-thin sections was prepared using a microtome and contrasted by means of 4% uranyl acetate-staining for 10 min and rinsed with water. Images were taken using JOEL JEM 2100F transmission electron microscope (Electron Microscopy Unit, University of Pretoria, Pretoria, South Africa) [23].

### Scanning electron microscopy

Scanning electron microscopy (SEM) was used to determine the effect of ESE-15-ol on the morphology on the cell surface [23]. Cells were seeded at 300,000 cells per well on heat-sterilized coverslips in 6-well plates. After 24 h, and to allow for attachment, cells were exposed to the compounds (2ME2, ESE-15-ol) and incubated for 24 h. Cells were fixed in 2.5% glutaraldehyde in 0.075 M phosphate buffer for 1 h and rinsed 3 times for 5 min each with 0.075 M phosphate buffer. Cells were fixed in 0.25% aqueous osmium tetroxide for 30 min and rinsed three times in distilled water in a fume cupboard. Samples were dehydrated with increasing concentrations of ethanol (30, 50, 70, 90, 100, 100 and 100%). Samples were covered in hexamethyldisilazane, left to dry in a dessicator overnight, sputter-coated in gold and viewed under a JSM 840 Scanning Electron Microscope JEOUL, Tokyo, Japan [23].

### Cell cycle progression

Cell cycle distribution and the detection of a sub-G_1_ apoptotic peak were analysed by flow cytometry using propidium iodide (PI) that stains deoxyribonucleic acid (DNA) [8]. HeLa cells (1,000,000) were seeded in 25 cm^2^ flask. After 24 h the medium was discarded and cells were exposed to 1 μM 2ME2 and 180 nM ESE-15-ol for 24 h in 5 ml, respectively. Cells were trypsinized and resuspended in 1 ml growth medium. Samples were centrifuged for 5 min at 300×*g* in order to pellet them. The supernatant was discarded and samples were resuspended in 200 μl of ice-cold phosphate buffer saline (PBS) containing 0.1% FCS. Ice-cold 70% ethanol (4 ml) was added in a drop wise manner on a vortex in order to avoid cell clumping. Samples were stored at 4 °C for 24 h. After 24 h cells were pelleted by centrifuging the cells at 300×*g* for 5 min. Supernatant was removed and cells were resuspended in 1 ml of PBS containing 40 μg/ml PI, 0.1% triton X-100 and 100 μg/ml RNase A. The solution was incubated at 37 °C, 5% CO_2_ for 45 min. PI fluorescence (relative DNA content per cell) was measured with a FC500 System flow cytometer [Beckman Coulter South Africa (Pty) Ltd.] equipped with an air-cooled argon laser excited at 488 nm. Data from at least 10,000 cells were analyzed with CXP software [Beckman Coulter South Africa (Pty) Ltd]. Data from cell debris (particles smaller than apoptotic bodies) and clumps of two or more cells were removed from further analysis. Cell cycle distribution was generated from the histograms by the Cyflogic version 1.2.1 software (Pertu Therho, Turko, Finland). Results were expressed as a percentage of the cells in each phase.

### Annexin V-FITC/propidium iodide staining

Apoptotic cells have exposed phosphatidylserine molecules that bind annexin V, while necrotic cells have compromised membranes and thus take up PI. Four different populations of cells were distinguished: those that were unlabelled (viable cells), those that have bound annexin V-FITC only (early apoptotic), those that have been stained with PI (necrotic) and those that have both bound annexin V and been labelled with PI (late apoptotic/necrotic cells) [24]. Analysis was performed using the BioVision Annexin V-FITC reagent kit acquired from BioVision Research Products (Mountain view, California, USA). The 1X binding buffer (supplied in the kit) was prepared as directed. HeLa cells (1,000,000) were seeded in 25 cm^2^ flask. After 24 h, the medium was discarded and the cells were exposed to 1 μM 2ME2 and 180 nM ESE-15-ol for 24 h in 5 ml, respectively. Cells were trypsinized and resuspended in 1 ml PBS for washing and samples were centrifuged at 300×*g*. The supernatant collected was discarded and the pellet was again washed twice with 1 ml of 1X binding buffer, and centrifuged at 300×*g* for 10 min. Cells were than resuspended in 100 μl of 1X binding buffer. Staining was done by adding annexin V-FITC (10 μl) for 15 min room temperature in the dark. Cells were washed by adding 1 ml of 1X binding buffer and thereafter cells were centrifuged at 300×*g* for 10 min. Cells were resuspended in 500 μl of 1X binding buffer solution. Subsequently, 5 μl of PI (100 µg/ml) was added and samples were gently mixed. Annexin V (FL1) and PI (FL3) fluorescence were measured in 10,000 cells per sample with a FC500 System flow cytometer [Beckman Coulter South Africa (Pty) Ltd.] equipped with an air-cooled argon laser excited at 488 nm. Results were analysed in Cyflogic version 1.2.1 software (Pertu Therho, Turko, Finland) statistics and expressed as a percentage of cells in three categories namely; viable cells, early apoptotic cells, late apoptosis and necrotic cells.

### Caspase 8 activity

Apoptosis is a controlled process resulting in cell death that relies on caspase-dependent proteolytic cascade resulting in the demise of the cell. Apoptosis is generally categorized as one of three modes namely, intrinsic (also known as the mitochondrial pathway), extrinsic (also known as death receptor pathway) and the endoplasmic pathway [25, 26]. The intrinsic pathway is triggered by irradiation, starvation and chemicals and takes part via mitochondrial involvement. It is characterised by mitochondrial outer membrane permeabilization and activation of initiator caspase 9 [26, 27]. The endoplasmic reticulum pathway is independent of the mitochondria. Any stress including oxidative stress, accumulation of misfolded proteins leads to endoplasmic reticulum stress that possibly results in apoptosis via caspase 12 [28, 29]. The extrinsic apoptosis pathway is initiated by the binding of a death ligand to a death receptor resulting in the activation of initiator caspase 8 [30]. The activation of caspase 8 was determined using a FLICE/caspase 8 colorimetric kit [31]. HeLa cells (1,000,000) were seeded in 25 cm^2^ flask. After 24 h the medium was discarded and cells were exposed to 1 μM 2ME2 and 180 nM ESE-15-ol for 24 h in 5 ml, respectively. After 24 h exposure to 1 μM 2ME2 and 180 nM ESE-15-ol cells were detached with trypsin and centrifuged at 13,000×*g*. Cells were resuspended in 50 µl of chilled cell lysis buffer and incubated on ice for 10 min. Cells were centrifuged at 10,000×*g* for 1 min. Supernatant was transferred to a fresh tube and put on ice. After determination the protein concentration using the bicinchoninic acid (BCA) protein assay (Thermo Fisher Scientific, Johannesburg, South Africa), 100 µg protein/50 µl cell lysis buffer was mixed with 50 µl 2X reaction buffer [containing 10 mM Dithiothreitol (DTT)]. The caspase-specific substrate, 5 µl 4 mM Ac-Ile-Glu-Thr-Asp-p-nitroanilide (Ac-IETD-pNA) was added and the mixture was incubated at 37 °C for 120 min (200 µM final concentration). Absorbance was determined at 405 nm on the EL×800 Universal Microplate Reader available from Bio-Tek Instruments Inc. (Vermont, USA).

### Statistical analysis of data

Data were obtained from at least three biological repeats for all experiments. Quantitative data consisted out of cell cycle analysis, annexin V-FITC and caspase 8 assessments. Qualitative data were obtained by means of PlasDIC, transmission electron microscopy and SEM. Quantitative data were statistically analyzed for significance using the Student’s *t* test*. P* values of <0.05 were regarded as statistically significant. Means were represented in bar charts with T-bars referring to standard deviations. Qualitative experiments were repeated at least thrice where data was obtained from PlasDIC, TEM and SEM. Regarding flow cytometry data from at least 10,000 events was analyzed using Cyflogic 1.2.1 software (Pertu Terho & Cyflo Ltd.).

## Results

### Polarization-optical transmitted light differential interference contrast

**Fig. 1:**
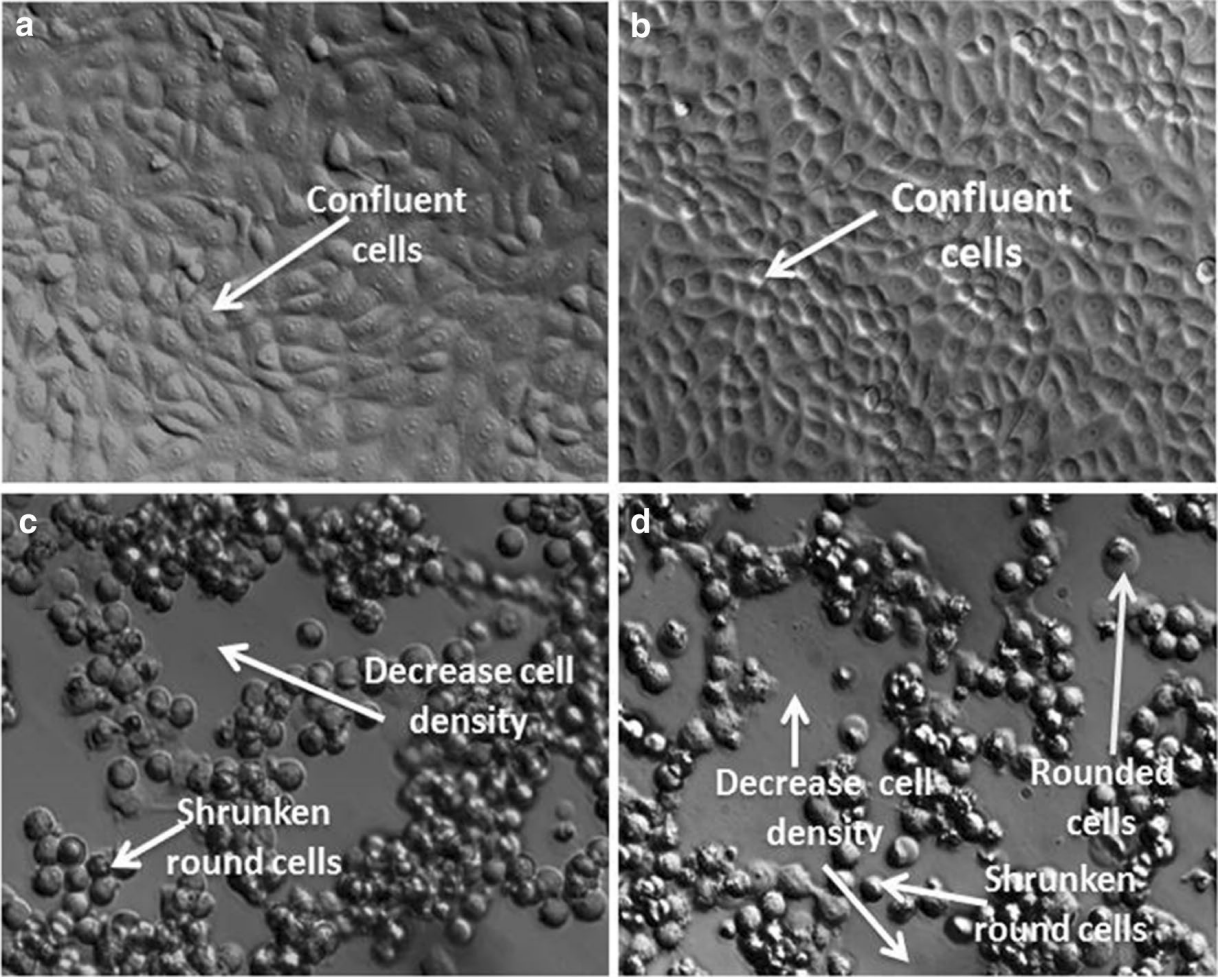
PlasDIC micrographs demonstrating effects on cell density and cell rounding. PlasDIC micrographs of HeLa cells after 24 h exposure at 20× magnification **a** HeLa cells propagated in growth medium and **b** vehicle-treated control exposure demonstrated confluent HeLa cells. HeLa cells exposed to **c** 180 nM ESE-15-ol and **d** 1 μM 2ME2 for 24 h results demonstrated a reduction in cell density and rounded shrunken cells

PlasDIC was used to view morphology after exposure to 180 nM ESE-15-ol and 1 µM 2ME2 for 24 h in HeLa cells when compared to cells propagated in growth medium and vehicle-treated cells (Fig. 1). These concentrations were used since previous studies conducted in our laboratory demonstrated optimal antiproliferative activity at these respective concentrations. ESE-15-ol and 2ME2-treated HeLa cells demonstrated cell rounding and reduced cell density when compared to cells propagated in growth medium and 0.024% vehicle-treated cells.

### Transmission electron microscopy

**Fig. 2:**
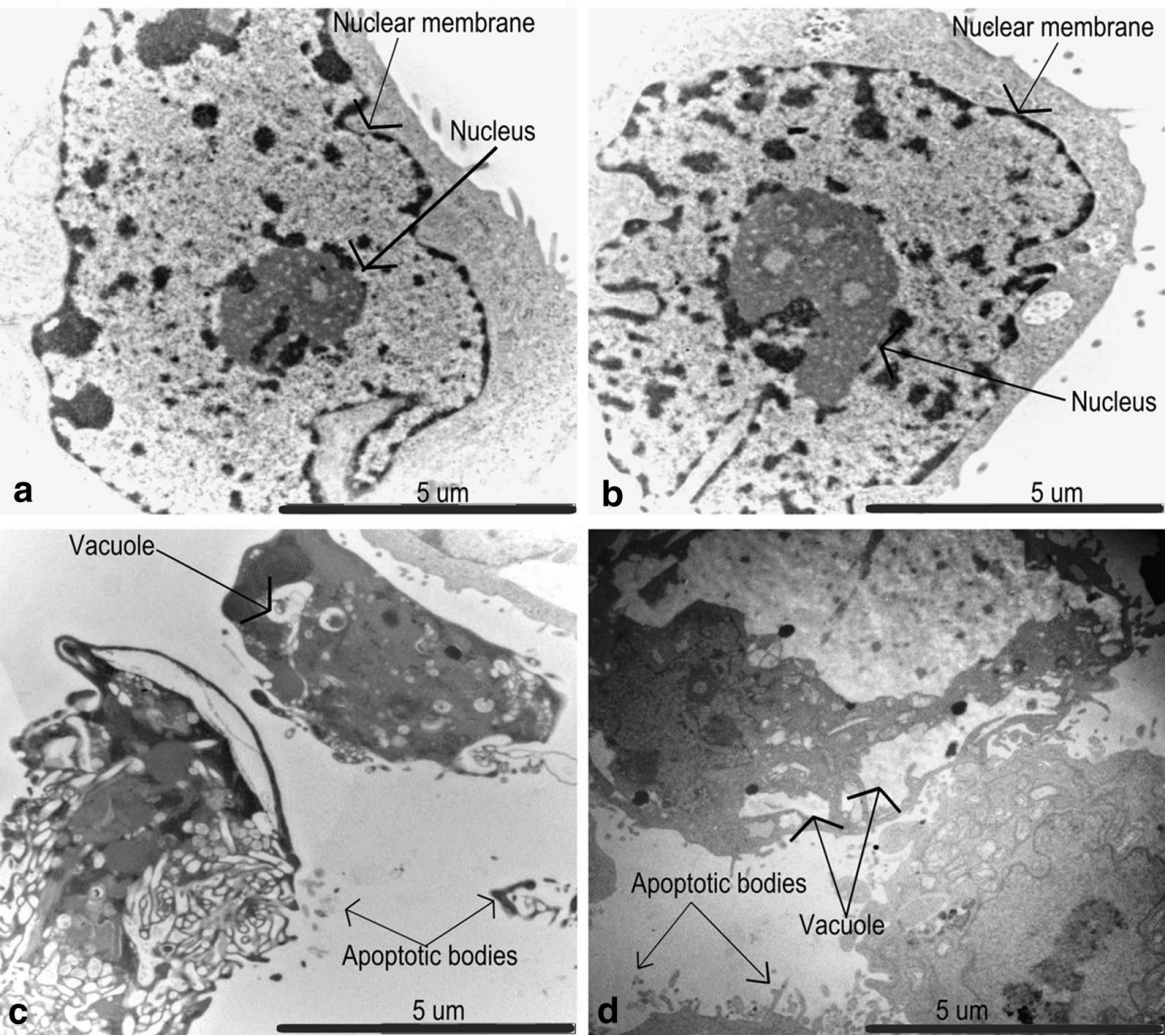
Transmission electron microscopy showing the internal cellular structure. Transmission electron microscopy of HeLa cells at (5 μm × 12,000×) magnification propagated in growth medium (**a**) and vehicle-treated (**b**) represented intact nuclear membrane. The 180 nM ESE-15-ol-treated cells (**c**) and 1 μM 2ME2-treated cells (**d**) after exposure for 24 h demonstrated apoptotic bodies and vesicle formation

Transmission electron microscopy was used to demonstrate effects on the internal cellular structure after exposure to 180 nM ESE-15-ol and 1 μM 2ME2 on HeLa cells (Fig. 2). HeLa cells propagated in growth medium and vehicle-treated control cells exhibited normal nuclear membrane and cytoplasm, with no sign of distress. ESE-15-ol and 2ME2 exposure resulted in the formation of vacuoles and apoptotic bodies.

### Scanning electron microscopy

**Fig. 3:**
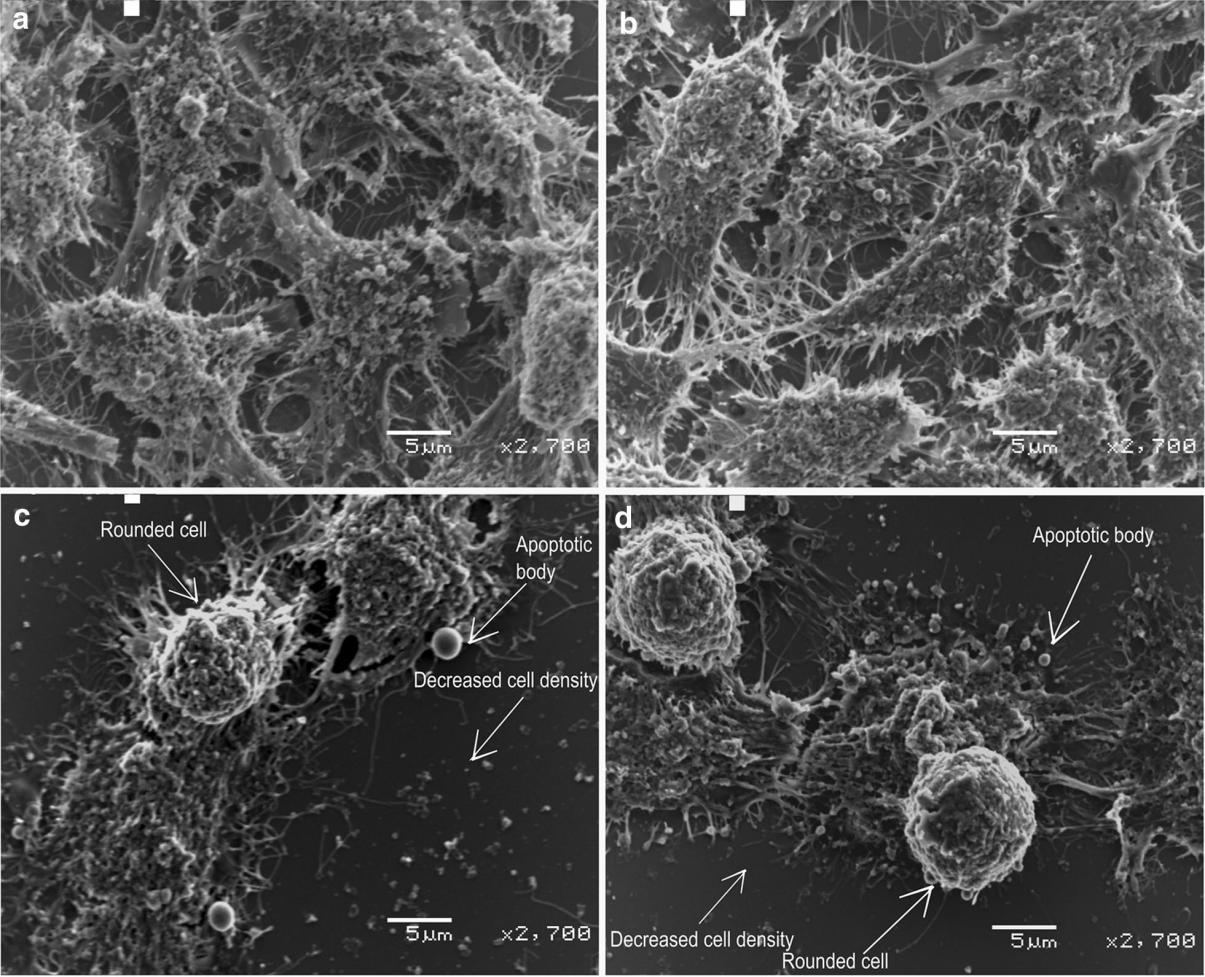
Scanning electron microscope demonstrating effects on the cell surface. Scanning electron microscopy of HeLa cells after exposure to growth medium (**a**) and DMSO vehicle-treated (**b**) control cells exposure demonstrated confluent growth of HeLa cells. Cells exposed to ESE-15-ol (**c**) and 2ME2 (**d**) demonstrated decreased cell density, rounded cells and apoptotic bodies

SEM was used to show the effects of 180 nM ESE-15-ol and 1 μM 2ME2 on the cell surface morphology. Cells propagated in growth medium and vehicle-treated cells were confluent with no signs of distress (Fig. 3). Cells exposed to ESE-15-ol and 2ME2 demonstrated decreased cell density, rounded cells and apoptotic bodies.

### Cell cycle progression

**Fig. 4:**
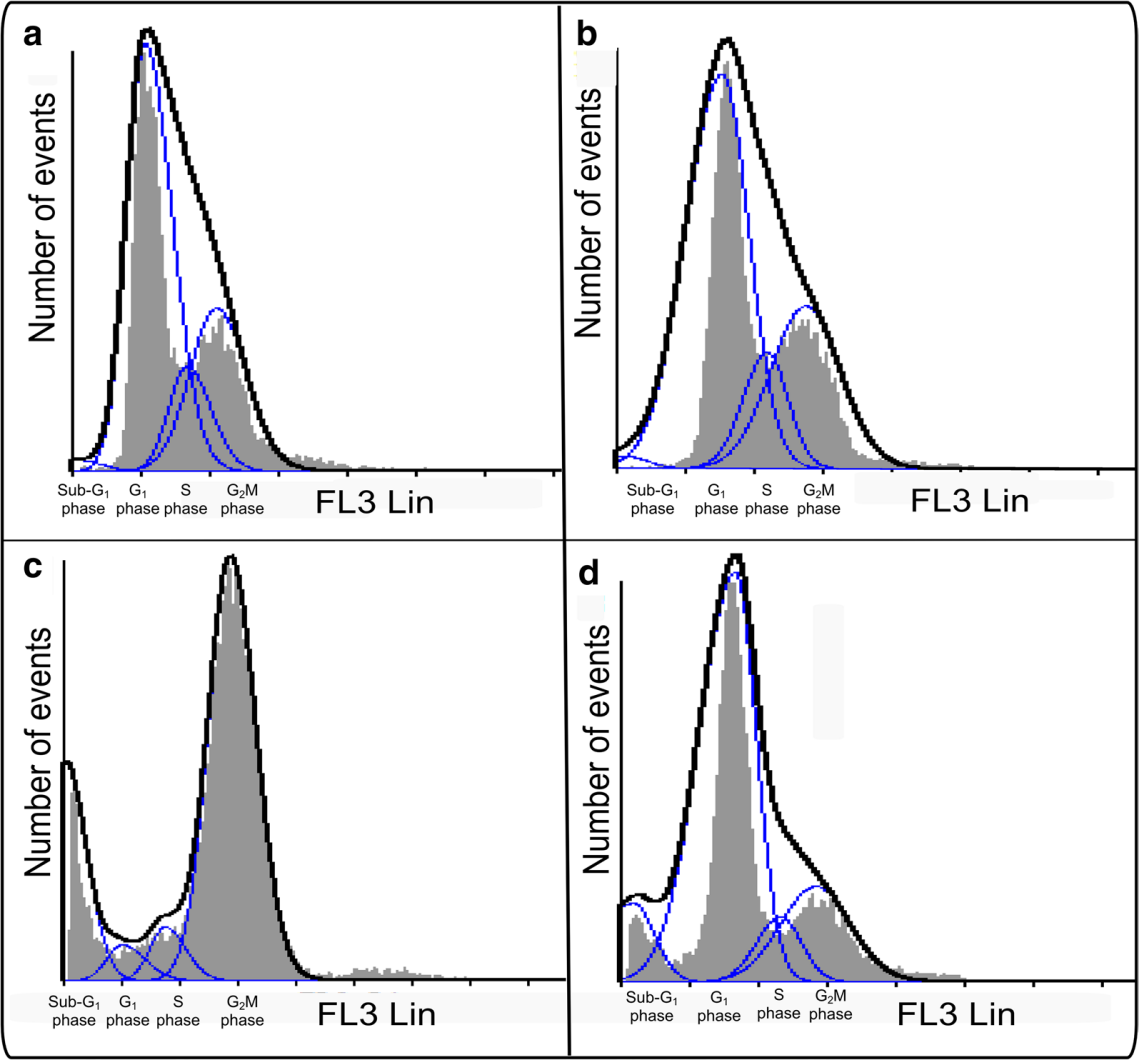
Histograms of ethanol fixation and PI staining used for cell cycle progression. Cell cycle analyses of cells propagated in growth medium (**a**), 0.024% vehicle-treated cells (**b**), 180 nM ESE-15-ol-treated cells (**c**) and 1 μM 2 ME2-treated cells (**d**). The sub-G_1_ population increased to 17% when exposed to ESE-15-ol and 7% after exposure to 2ME2. In addition, exposure to ESE-15-ol resulted in an increased number of cells occupying G_2_M phase (72%) when compared to vehicle-treated cells and cells propagated in growth medium

**Fig. 5:**
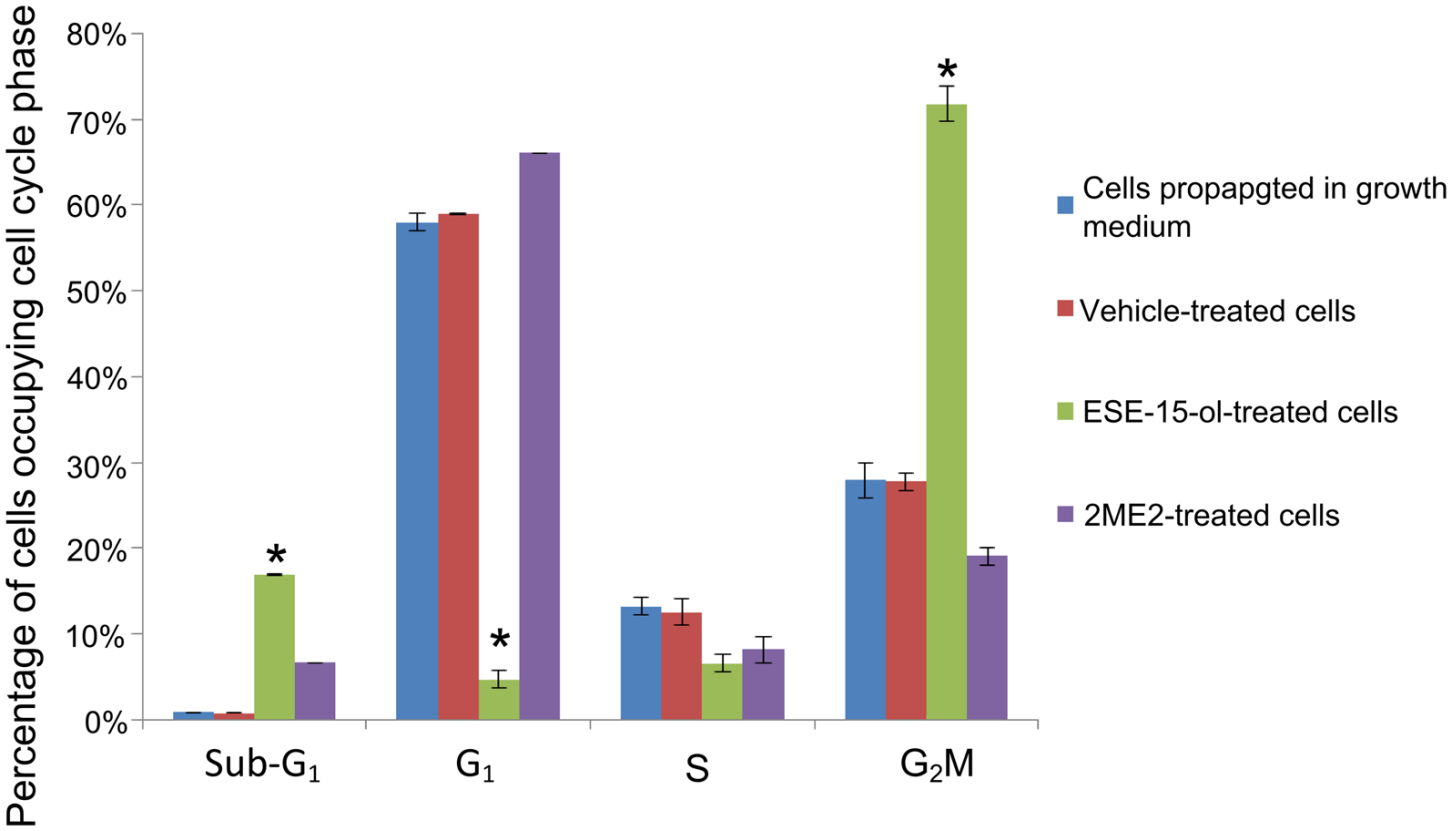
Percentage of cells occupying each cell cycle phase. Cell cycle progression of cells propagated in growth medium, 0.024% vehicle-treated cells, 180 nM ESE-15-ol-treated cells and 1 μM 2ME2-treated cells. Exposure to both ESE-15-ol and 2ME2 resulted in an increased sub-G1 peak. However, only exposure to ESE-15-ol resulted in a significant increase in cells occupying the G_2_M phase (an *asterisk* indicates *P*  < 0.05 when compared to 2ME2-treated cells)

Flow cytometry was used to determine the effect of 180 nM ESE-15-ol and 1 μM 2 ME2 on cell cycle progression (Figs. 4, 5; Table 1). Results were calculated as percentage cells in each stage of the cell cycle. Cell cycle distribution of the cells propagated in growth medium revealed an average of 0.88% in sub-G_1_ population, 58% in the G_1_ phase, 13% in the S phase and 28% in the G_2_M phase. The vehicle-treated sample revealed an average of 0.75% in sub-G_1_ population, 59% in the G1 phase, 13% in the S phase and 28% in the G_2_M phase representing a cell population in logarithmic growth. A statistically significant increase of 17% cells in sub-G_1_ was demonstrated in ESE-15-ol treated cells. ESE-15-ol-treated cells showed a decrease in G_1_-(5%) and S phase (7%) and a distinct increase of cells in the G_2_M phase (72%) indicating an increase in cells present in a metaphase block. 2ME2-treated cells presented with 7% cells in sub-G_1_, 66% cells in G_1_ phase, 8% cells in S phase and 19% of cells in G_2_M phase.

**Table 1:** Cell cycle progression using flow cytometry, ethanol fixation and PI staining

	Sub-G_1_ (%)	G_1_ (%)	S (%)	G_2_M (%)
Cells propagated in growth medium	0.88	58	13	28
Vehicle-treated cells	0.75	59	13	28
ESE-15-ol-treated cells	17*	5*	7	72*
2ME2-treated cells	7	66	8	19

* Indicates *P* < 0.05 when compared to 2ME2-treated cells

### Annexin V-FITC/propidium iodide staining

**Fig. 6:**
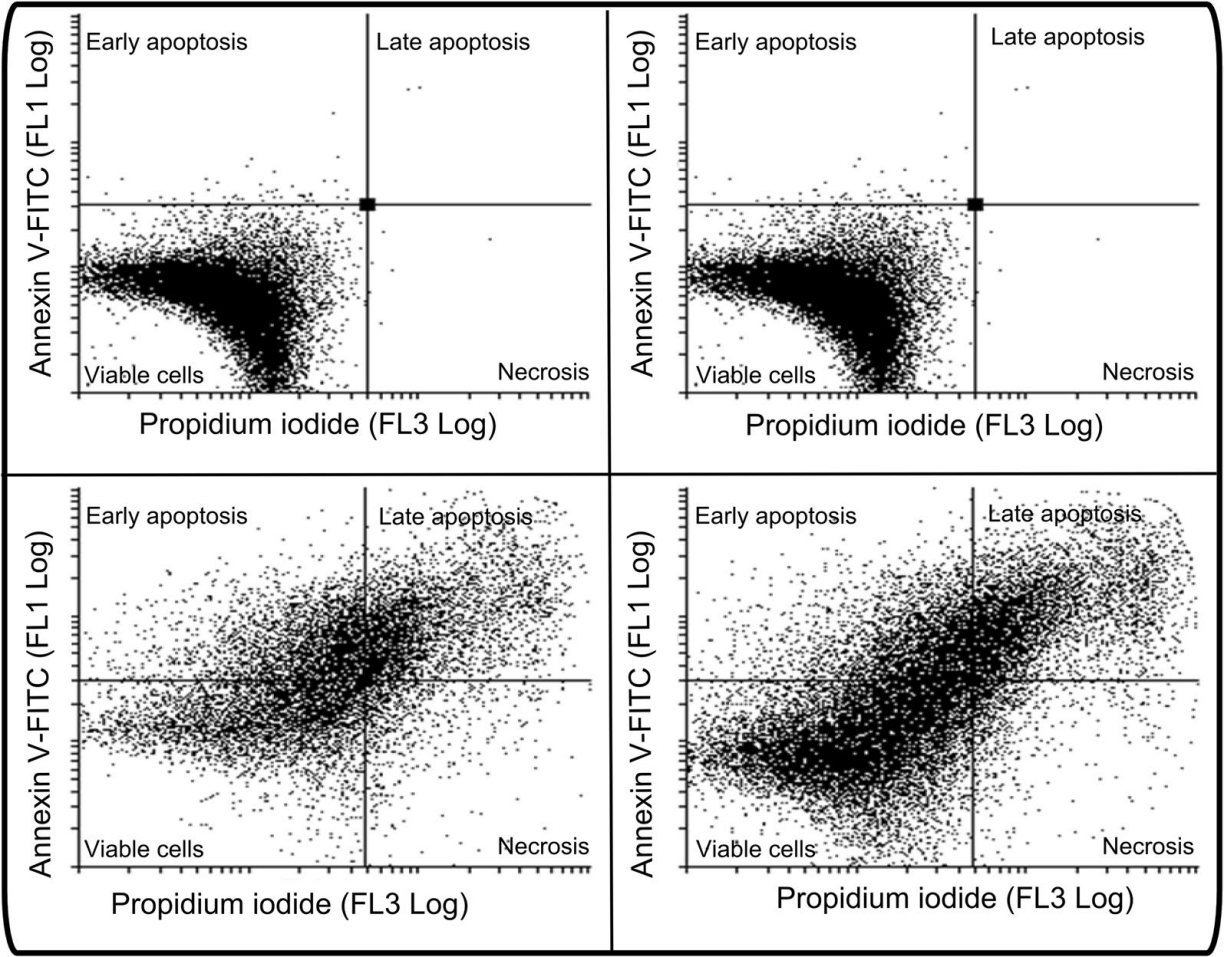
Annexin V-FITC and PI staining showing apoptosis induction. Flow cytometry using annexin V-FITC and PI staining of cells propagated in growth medium (**a**), 0.024% vehicle-treated control (**b**), 180 nM ESE-15-ol-treated cells (**c**) and 1 μM 2ME2-treated cells (**d**). Exposure to ESE-15-ol and 2ME2 resulted in significant loss of viability (*P* < 0.05). ESE-15-ol-treated cells presented with 25% viable cells, 41% early apoptotic cells, 15% late apoptotic cells and 19% necrotic cells. 2ME2-treated cells presented with 48% viable cells, 26% early apoptotic cells, 15% late apoptotic cells and 11% necrotic cells

The presence of cells in apoptosis was investigated by means of flow cytometry and Annexin V-FITC (Fig. 6; Table 2). HeLa cells propagated in growth medium and 0.024% vehicle-treated cells showed intact cell membrane and no sign of apoptosis with 97% being viable. HeLa cells exposed to ESE-15-ol showed decreased cell viability (25%) accompanied with an increase in cells present in early apoptotic cells (41%), late apoptotic cells (15%) and necrotic cells (19%). 2ME2-treated cells presented with 48% viable cells, 26% early apoptotic cells, 15% late apoptotic cells and 11% necrotic cells.

**Table 2:** Flow cytometry using annexin V-FITC demonstrating viable cells, cells in early apoptosis, cells in late apoptosis and necrotic cells

	Viable cells (%)	Early apoptosis (%)	Late apoptosis (%)	Necrosis (%)
Cells propagated in growth medium	98	0.98	0	1
Vehicle-treated cells	98	1	0	1
ESE-15-ol-treated cells	25*	41*	15	19*
2ME2-treated cells	48	26	15	11

* Indicates *P* < 0.05 when compared to 2ME2-treated cells

### Caspase 8 activity

**Fig. 7:**
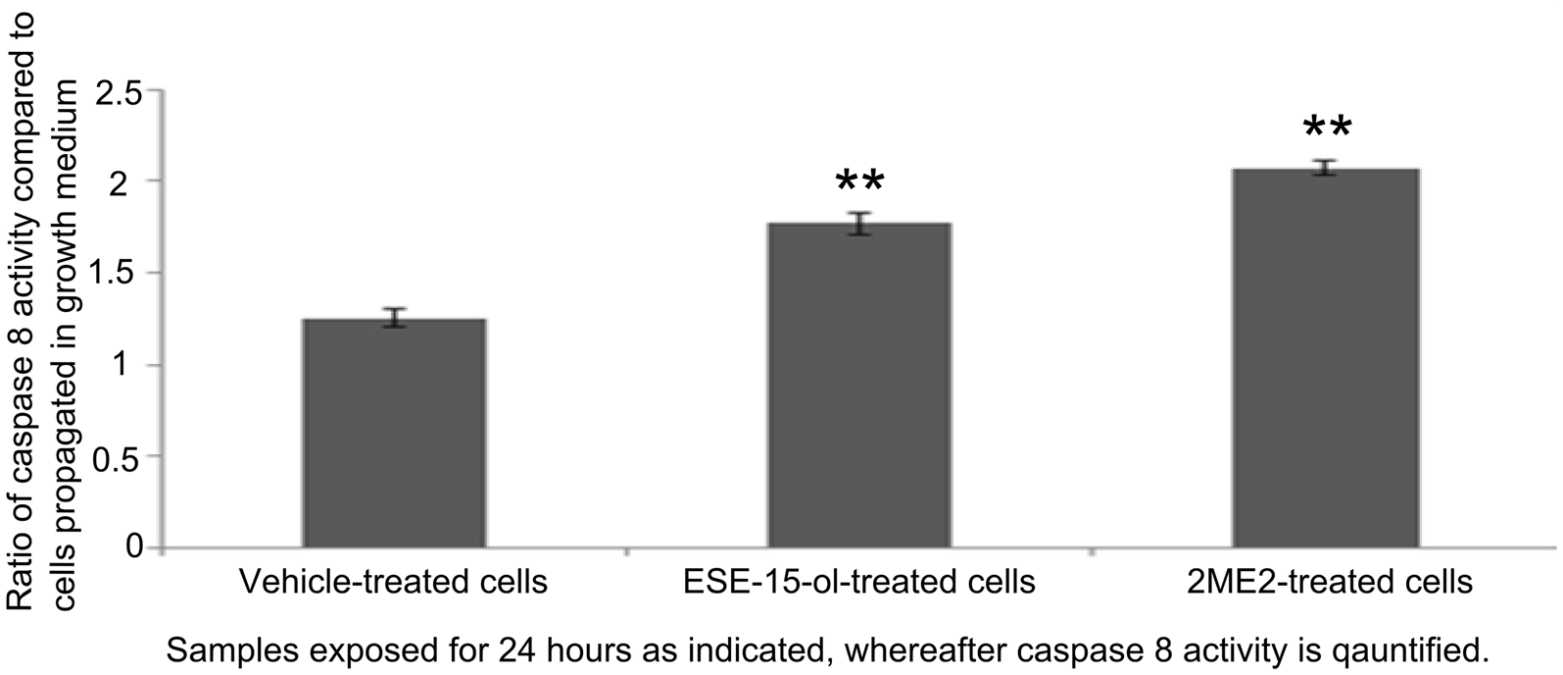
Caspase 8 activity measured by spectrophotometry. Caspase 8 activity ratios of ESE-15-ol- and 2ME2-treated HeLa cells compared to cells propagated in growth medium only after 24 h of exposure. Caspase 8 activities were statistically significantly increased in the ESE-15-ol- and-2 ME2-treated cells when compared to vehicle-treated cells and cells propagated growth medium. An *double asterisk* indicates *P* < 0.05 when compared to cells propagated in growth medium and vehicle-treated cells

Influence of 180 nM ESE-15-ol and 1 μM 2ME2 (24 h) on caspase 8 activity was investigated by means spectrophotometry (Fig. 7). The ratio of caspase 8 activities was measured with reference to cells propagated in growth medium. There was no statistically significant difference with regard to caspase 8 activity in vehicle-treated cells when compared to cells propagated in growth medium. The ESE-15-ol-treated sample showed a 1.7-fold increase in caspase 8 activity when compared to cells propagated in growth medium. 2ME2-treated sample showed twofold increase compared to cells propagated in growth medium (Fig. 7).

## Discussion

Computer modelling techniques benefit the drug development industry since this approach reduces the cost of drug development by 50% [32]. In silico-design process takes into account parameters such as activity, absorption, distribution, metabolism and excretion and has resulted in a rational drug identification tool [33]. However, in silico-design methods are still computer-based and can’t be directly extrapolated to the efficacy of the compound. Computer-based technologies rely on algorithms that utilize perfect standards and they are not comparable to the biological complexities [34]. Thus, in vitro assessments laboratory testing of these in silico-designed compounds are essential. This study thus evaluated whether an in silico-designed compound (ESE-15-ol) is as effective as docking- and receptor modelling predicts.

Previous research has revealed that 2ME2 plays a crucial role in the induction of apoptosis [8, 35–37]. Due to the limited metabolic accessibility and rapid degradation of 2ME2, research in our laboratory in silico-designed and subsequently synthesized novel 2ME2 analogues based on the notion that sulphamate addition increased oestrogenic bioavailability by avoiding hepatic first-pass hepatic metabolism [38]. Therefore, a 2ME2 analogue, ESE-15-ol was in silico-designed in our laboratory [3]. Dose-dependent studies were conducted in our laboratory and the optimal antiproliferative activity in HeLa cells were established at 180 nM of ESE-15-ol (data not shown). Several additional 2ME2 analogues exerted antiproliferative activity within this concentration range (200 nM–1 µM) in cell lines including the MCF-7 cell line and esophageal carcinoma SNO cells [11, 39].

Morphological studies of cells exposed to 180 nM ESE-15-ol and 1 μM 2ME2 for 24 h showed decreased cell density, apoptotic bodies, ghost cells, rounded and shrunken cells and cell debris. Previous studies indicated that morphological characteristics of exposure to another 2ME2 analogue, namely 2-ethyl-3-*O*-sulphamoyl-estra-1,3,5(10)16-tetraene (ESE-16) included cell shrinkage, membrane blebbing condensation of nuclear chromatin into sharply delineated masses that become marginated against the nuclear membrane, vacuolar formation, shrunken round cells, decreased cell density, cell debris, cell distress as well as formation of apoptotic bodies [11]. Sulphamoylated 2ME2 analogues exposed to HeLa cells and 2-methoxyestradiol-bis-sulphamate exposed in MCF-7 cells showed in vitro cytotoxic effect, but did not reflect possible damage to cell membrane integrity [38]. Thus, according to morphological evidence ESE-15-ol behave in a similar manner to 2ME2, but at a pharmacological concentration indicating an improved bioavailability.

Cell cycle progression analysis of ESE-15-ol-treated HeLa cells showed a G_2_M block, as well as increase in sub-G_1_ fraction when compared to growth medium and vehicle-treated control cells and 2ME2. Exposure of ovarian and prostate cancer cells to another sulphamoylated 2ME2 analogue, namely 2-methoxyestradiol-bis-sulphamate and ESE-16-exposed MDA-MB-231 cells and ESE-16-exposed HeLa cells for 24 h showed an increase in both G_2_M block and sub-G_1_ fraction. After 48 h, nearly all the cells were present in the sub-G_1_ fraction [5, 38, 39]. Exposure to both ESE-15-ol and 2ME2 resulted in a decreased percentage of cells occupying the S phase. A number of intra-S-phase-related checkpoint proteins involved in DNA repair were downregulated in 2ME2-treated MCF-7 cells which indicated that cells are most likely to re-enter mitosis cycle [10].

Flow cytometry study using annexin V-FITC and PI demonstrated that ESE-15-ol and 2ME2 resulted in the induction of apoptosis. However, the apoptotic activity exerted by ESE-15-ol is more prominent at a pharmacological concentration of 180 nM when compared to the apoptotic activity induced by 2ME2 at physiological concentration of 1 μM. Theron et al. [11] reported that HeLa cells exposed to ESE-16 showed loss of cell membrane asymmetry occurring with the translocation of phosphatidylserine to the outside of the cell during apoptosis induction [11]. 2ME2 has been implicated in induction of the apoptotic pathway in several cell lines [39, 40]. Similar to our study, ESE-16-treated HeLa cells at 0.5 μM also showed upregulation in caspase 8 activity [38]. The analysis of caspase 8 activity demonstrated a twofold increase in 1 μM 2ME2-treated cells and a 1.7-fold increase in 180 nM ESE-15-ol-treated cells. This study is the first to indicate that the novel in silico-designed estradiol analogue, ESE-15-ol, may act with improved potency as a potential anticancer agent when compared to 2ME2 at a lower dose. Both the source molecule and its analogue resulted in a statistically significant increase in caspase 8 activity demonstrating the involvement of apoptosis.

## Conclusion

The role of computational biology is becoming increasingly important in drug design. It remains challenging to accurately predict pharmacological parameters including activity and toxicology of these in silico-designed compounds. By combining structural information and in vitro studies a predictive tool on possible biological responses are provided [41]. Data from this in vitro study supports the concept that ESE-15-ol induced apoptosis in cervical adenocarcinoma (HeLa) cells in response to an increase in the G_2_M block accompanied with increased caspase 8 activity demonstrating the induction of the apoptotic pathway. In this study it was also demonstrated that ESE-15-ol exerted increased potent anticancer activity when compared to 2ME2. This confirms that in silico-designed identification is effective to assess mechanism of action of in silico-designed potential anticancer compounds of cell death induction via apoptosis.

## References

[1] De la Cruz-Hernández E, Pérez-Cárdenas E, Contreras-Paredes A, Cantú D, Mohar A, Lizano M (2007). The effects of DNA methylation and histone deacetylase inhibitors on human papillomavirus early gene expression in cervical cancer, an in vitro and clinical study. Virol J.

[2] de Bree E, Theodoropoulos PA, Rosing H, Michalakis J, Romanos J, Beijnen JH, Tsiftsis DD (2006). Treatment of ovarian cancer using intraperitoneal chemotherapy with taxanes: from laboratory bench to bedside. Cancer Treat Rev.

[3] Stander BA, Joubert F, Tu C, Sippel KH, McKenna R, Joubert AM (2012). In vitro evaluation of ESE-15-ol, an estradiol analogue with nanomolar antimitotic and carbonic anhydrase inhibitory activity. PLoS ONE.

[4] Zhou J, Giannakakou P (2005). Targeting microtubules for cancer chemotherapy. Curr Med Chem Agents.

[5] Stander A, Joubert F, Joubert A (2011). Docking, synthesis, and in vitro evaluation of antimitotic estrone analogs. Chem Biol Drug Des.

[6] Hsieh CC, Kuo YH, Kuo CC, Chen LT, Cheung CH, Chao TY (2010). Chamaecypanone C, a novel skeleton microtubule inhibitor, with anticancer activity by trigger caspase 8-Fas/FasL dependent apoptotic pathway in human cancer cells. Biochem Pharmacol.

[7] Chua YS, Chua YL, Hagen T (2010). Structure activity analysis of 2-methoxyestradiol analogues reveals targeting of microtubules as the major mechanism of antiproliferative and proapoptotic activity. Mol Cancer Ther.

[8] Visagie MH, Joubert AM (2011). In vitro effects of 2-methoxyestradiol-bis-sulphamate on reactive oxygen species and possible apoptosis induction in a breast adenocarcinoma cell line. Cancer Cell Int.

[9] Matei D, Schilder J, Sutton G, Perkins S, Breen T, Quon C (2009). Activity of 2 methoxyestradiol (Panzem NCD) in advanced, platinum-resistant ovarian cancer and primary peritoneal carcinomatosis: a hoosier oncology group trial. Gynecol Oncol.

[10] Stander BA, Marais S, Vorster CJJ, Joubert AM (2010). In vitro effects of 2-methoxyestradiol on morphology, cell cycle progression, cell death and gene expression changes in the tumorigenic MCF-7 breast epithelial cell line. J Steroid Biochem Mol Biol.

[11] Theron AE, Nolte EM, Lafanechère L, Joubert AM (2013). Molecular crosstalk between apoptosis and autophagy induced by a novel 2-methoxyestradiol analogue in cervical adenocarcinoma cells. Cancer Cell Int.

[12] Kapetanovic IM (2008). Computer-aided drug discovery and development (CADDD): in silico-chemico-biological approach. Chem Bio Interact.

[13] Li Y, Wang H, Oosterwijk E, Tu C, Shiverick KT, Silverman DN (2009). Expression and activity of carbonic anhydrase IX is associated with metabolic dysfunction in MDA-MB-231 breast cancer cells. Cancer Invest.

[14] Ward C, Meehan J, Mullen P, Supuran C, Dixon JM, Thomas JS (2015). Evaluation of carbonic anhydrase IX as a therapeutic target for inhibition of breast cancer invasion and metastasis using a series of in vitro breast cancer models. Oncotarget.

[15] Repsold L, Mqoco T, Wolmarans E, Nkandeu S, Theron J, Piorkowski T, du Toit P, van Papendorp P, Joubert AM (2014). Ultrastructural changes of erythrocytes in whole blood after exposure to prospective in silico-designed anticancer agents: a qualitative case study. Biol Res.

[16] Rahbari R, Sheahan T, Modes V, Collier P, Macfarlane C, Badge RM (2009). A novel L1 retrotransposon marker for HeLa cell line identification. Biotechniques.

[17] Visagie MH, Mqoco TV, Liebenberg L, Mathews EH, Mathews GE, Joubert AM (2015). Influence of partial and complete glutamine- and glucose deprivation of breast- and cervical tumorigenic cell lines. Cell Biosci.

[18] Laundry JJ, Pyl PT, Rausch T, Zichner T, Tekkedil MM, Stütz AM (2013). The genomic and transcriptomic landscape of a heLa cell line. G3 (Bethesda).

[19] Adey A, Burton JN, Kitzman JO, Hiatt JB, Lewis AP, Martin BK (2013). The haplotype-resolved genome and epigenome of the aneuploidy HeLa cancer cell line. Nature.

[20] Visagie MH, Joubert AM (2011). 2-Methoxyestradiol-bis-sulfamate induces apoptosis and autophagy in a tumorigenic breast epithelial cell line. Mol Cell Biochem.

[21] Esslinger M, Gross H (2015). Simulation of differential interference contrast microscopy and influence of abberrations. J Microsc.

[22] Visagie MH, Joubert AM (2010). The in vitro effects of 2-methoxyestradiol-bis-sulphamate on cell numbers, membrane integrity and cell morphology, and the possible induction of apoptosis and autophagy in a non-tumorigenic breast epithelial cell line. Cell Mol Biol Lett.

[23] Visagie MH, Birkholtz LM, Joubert AM (2014). 17-Beta-estradiol analog inhibits cell proliferation by induction apoptosis in breast cell lines. Microsc Res Tech.

[24] Vermes I, Haanen C, Steffens-Nakken H, Reutellingsperger C (1995). A novel assay for apoptosis flow cytometric detection of phosphatidylserine expression on early apoptotic cells using fluorescein labelled Annexin V. J Immunol Methods.

[25] Ashkenazi A (2015). Targeting the extrinsic apoptotic pathway in cancer: lessons learned and future directions. J Clin Invest.

[26] Wong RS (2011). Apoptosis in cancer: apoptosis in cancer: from pathogenesis to treatment. J Exp Clin Cancer Res.

[27] Chipuk JE, Bouchier-Hayes L, Green DR (2006). Mitochondrial outer membrane permeabilization during apoptosis: the innocent bystander. Cell Death Differ.

[28] Rao RV, Ellerby HM, Bredensen DE (2004). Coupling endoplasmic reticulum stress to the cell death program. Cell Death Differ.

[29] Groenedyk J, Mickalak M (2005). Endoplasmic reticulum quality control and apoptosis. Acta Biochim Pol.

[30] Fulda S (2015). Targeting extrinsic apoptosis in cancer: challenges and opportunities. Semin Cell Dev Biol.

[31] Visagie MH, Birkholtz LM, Joubert AM (2015). A 2-methoxyestradiol bis-sulphamoylated derivative induces apoptosis in breast cell lines. Cell Biosci.

[32] Wang Y, Xing J, Xu Y, Zhou N, Peng J, Xiong Z (2015). In silico ADME/T modelling for rational drug design. Q Rev Biophys.

[33] Tian S, Wang J, Li Y, Li D, Hou T (2015). The application of in silico drug-likeness predictions in pharmaceutical research. Adv Drug Deliv Rev.

[34] De Smet R, Marchal K (2010). Advantages and limitations of current network interference methods. Nat Rev Microbiol.

[35] Lis A, Ciesielski MJ, Barone TA, Scott BE, Fenstermaker RA, Plunkett RJ (2004). 2-Methoxyestradiol inhibits proliferation of normal and neoplastic glial cells, and induces cell death, in vitro. Cancer Lett.

[36] Marais S, Mqoco T, Stander A, van Dirk P, Joubert A (2012). The in vitro effects of a sulphamoylated derivative of 2-Methoxyestradiol on cell number, morphology and alpha-tubulin disruption in cervical adenocarcinoma (HeLa) cells. Biomed Res.

[37] Lavallee TM, Zhan XH, Johnson MS, Herbstritt CJ, Swartz G, Williams MS (2003). 2-Methoxyestradiol up-regulates death receptor 5 and induces apoptosis through activation of the extrinsic pathway. Cancer Res.

[38] Visagie M, Theron A, Mqoco T, Vieira W, Prudent R, Martinez A (2013). Sulphamoylated 2-methoxyestradiol analogues induce apoptosis in adenocarcinoma cell lines. PLoS ONE.

[39] Mqoco T, Marais S, Joubert A (2013). 2-Methoxyestradiol-bis-sulphamate: a promising anticancer agent in an esophageal carcinoma (SNO) cell line. Biomed Res.

[40] Basu A, Castle VP, Bouziane M, Bhalla K, Haldar S (2006). Crosstalk between extrinsic and intrinsic cell death pathways in pancreatic cancer: synergistic action of estrogen metabolite and ligands of death receptor family. Cancer Res.

[41] Krejsa CM, Horvath D, Rogalski SL, Penzotti JE, Mao B, Barbosa F (2003). Predicting ADME properties and side effects: the BioPrint approach. Curr Opin Drug Discov Devel.

